# Poly (*N*-vinylpyrrolidone) modification mitigates plasma protein corona formation on phosphomolybdate-based nanoparticles

**DOI:** 10.1186/s12951-021-01140-8

**Published:** 2021-12-23

**Authors:** Youyi Yu, Behafarid Ghalandari, Guangxia Shen, Liping Wang, Xiao Liu, Aiting Wang, Sijie Li, Haiyang Xie, Xianting Ding

**Affiliations:** State Key Laboratory of Oncogenes and Related Genes, Institute for Personalized Medicine, School of Biomedical Engineering, Shanghai Jiao Tong University, Shanghai, 200030 China

**Keywords:** Phosphomolybdate, Zwitterionic polymer poly (*N*-vinylpyrrolidone), Plasma protein corona, Mass spectrometry

## Abstract

Phosphomolybdate-based nanoparticles (PMo_12_-based NPs) have been commonly applied in nanomedicine. However, upon contact with biofluids, proteins are quickly adsorbed onto the NPs surface to form a protein corona, which induces the opsonization and facilitates the rapid clearance of the NPs by macrophage uptake. Herein, we introduce a family of structurally homologous PMo_12_-based NPs (CDS-PMo_12_@PVP_x_(x = 0 ~ 1) NPs) capping diverse content of zwitterionic polymer poly (*N*-vinylpyrrolidone) (PVP) to regulate the protein corona formation on PMo_12_-based NPs. The fluorescence quenching data indicate that the introduction of PVP effectively reduces the number of binding sites of proteins on PMo_12_-based NPs. Molecular docking simulations results show that the contact surface area and binding energy of proteins to CDS-PMo_12_@PVP_1_ NPs are smaller than the CDS-PMo_12_@PVP_0_ NPs. The liquid chromatography-tandem mass spectrometry (LC–MS/MS) is further applied to analyze and quantify the compositions of the human plasma corona formation on CDS-PMo_12_@PVP_x_(x = 0 ~ 1) NPs. The number of plasma protein groups adsorption on CDS-PMo_12_@PVP_1_ NPs, compared to CDS-PMo_12_@PVP_0_ NPs, decreases from 372 to 271. In addition, 76 differentially adsorption proteins are identified between CDS-PMo_12_@PVP_0_ and CDS-PMo_12_@PVP_1_ NPs, in which apolipoprotein is up-regulated in CDS-PMo_12_@PVP_1_ NPs. The apolipoprotein adsorption onto the NPs is proposed to have dysoponic activity and enhance the circulation time of NPs. Our findings demonstrate that PVP grafting on PMo_12_-based NPs is a promising strategy to improve the anti-biofouling property for PMo_12_-based nanodrug design.

**Supplementary Information:**

The online version contains supplementary material available at 10.1186/s12951-021-01140-8.

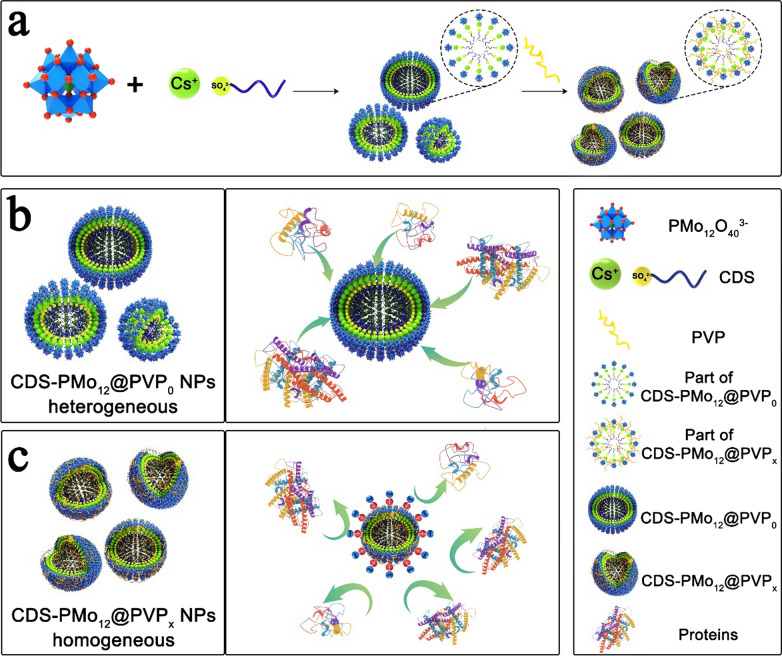

## Introduction

Phosphomolybdate (PMo_12_)-based nanocomposites and hybrids [1–7] have been reported with potentials for the treatment of vast types of cancers [8–13]. The nanoparticles (NPs) tend to undergo nonspecific protein adsorption (formation of “protein corona”) when entering the physiological system [14–16]. The “protein corona” complexes consist of dozens of proteins including apolipoproteins, adhesion mediators, signaling and transport proteins, and coagulation factors [17]. These native or conformational proteins act as opsonins that enable a nanomaterial for efficient uptake by the mononuclear phagocytes system (MPS) [18–20]. To reduce NPs clearance by the MPS, shielding groups on NPs surfaces are required to block opsonins proteins binding to NPs [18]. These groups tend to be long hydrophilic polymer chains and nonionic surfactants. Some polymers include polyethylene glycol (PEG), polysaccharides, polyacrylamide, and PEG-containing copolymers as examples of shielding groups. PEGylated Doxil nanodrug has been used as clinical medicine, yet PEG cannot completely shield protein adsorption [21] to modulate the immune response.

**Scheme 1:**
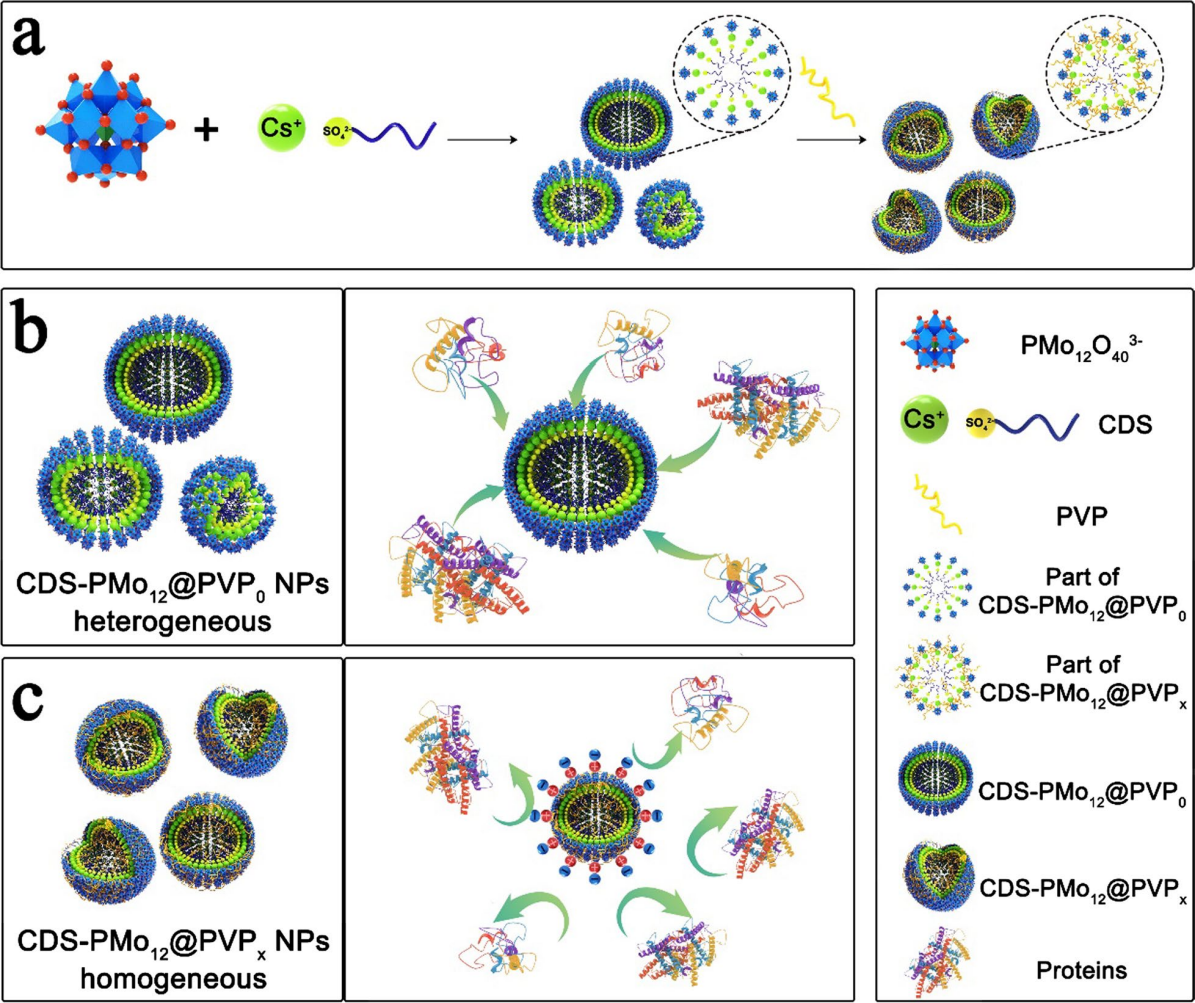
Preparation of CDS-PMo_12_@PVP_x_(x = 0 ~ 1) NPs for decreased adsorption of serum proteins. **a** CDS is used as a cationic nucleating agent to trap the PMo_12_O_40_^3−^ polyanion to synthesize CDS-PMo_12_@PVP_0_ NPs. The introduction of PVP to the CDS-PMo_12_O_40_^3−^ reaction system further synthesizes homogenous CDS-PMo_12_@PVP_x_(x = 0.05 ~ 1) NPs. **b** Heterogeneous CDS-PMo_12_@PVP_0_ NPs without PVP coating protection could largely adsorb proteins. **c** In contrast, PVP modified homogeneous CDS-PMo_12_@PVP_x_(x = 0.05 ~ 1) NPs could protect the NPs from protein adsorption

The poly(*N*-vinyl-2-pyrrolidone) (PVP) zwitterionic polymer is a sub-class of polyampholytes that possess equivalent positive and negative charges on the same pendant group maintaining overall electrical neutrality [22]. A correlation between surface charge and opsonization has been demonstrated in vitro, with research showing that neutrally charged NPs have a much lower opsonization rate than charged NPs [23]. In addition, studies on PMo_12_-based NPs mainly focus on the design and screening of potent PMo_12_-based nanomaterials [24, 25], as well as their pharmacology study [14, 26]. Limited effort has been devoted to the protein corona formation mechanism on zwitterionic polymer modified PMo_12_-based NPs. Herein, we synthesized a series of structurally homologous PMo_12_-based NPs (CDS-PMo_12_@PVP_x_(x = 0 ~ 1) NPs) capping diverse content of zwitterionic polymer poly (*N*-vinylpyrrolidone) (PVP) by micelle-based approach. The cesium dodecyl sulfate (C_12_H_25_SO_4_Cs, CDS) cationic surfactant is used to trap the PMo_12_O_40_^3−^ polyanion for preparing CDS-PMo_12_@PVP_0_ NPs (Scheme 1a) [25]. When the PVP is introduced to the reaction system, PVP firstly interacts with CDS to form CDS-PVP complex through the electrostatic/hydrophobic forces [27]. The CDS-PVP complex matrix decelerates the contact rate of the CDS and the PMo_12_O_40_^3−^ polyanion. Besides, the PVP polymer adhered to the surface of CDS-PMo_12_@PVP_x_ (x = 0.05 ~ 1) NPs serves as a protective layer to prevent the further aggregation of NPs. Consequently, the PVP that added to the reaction system manages to regulate the size and the surface properties of CDS-PMo_12_@PVP_x_(x = 0.05 ~ 1) NPs.

As a proof of concept, Cyt-C, Hb, and BSA were adopted as representative basic, neutral, and acidic proteins to investigate protein adhesion behaviors on CDS-PMo_12_@PVP_x_(x = 0 ~ 1) NPs. The adsorption efficiencies were gradually decreased when the PVP content increased, suggesting the addition of PVP suppresses the adsorption of protein to NPs (Scheme 1b, c). Fluorescence quenching measurements further unveiled the underlying interaction mechanism between proteins and two typical PMo_12_-based NPs (CDS-PMo_12_@PVP_x_(x = 0,1) NPs). The introduction of PVP influences the binding kinetics and thermodynamic process of protein adsorption, reducing the number of binding sites, and subsequently influences the adsorption of protein. The hydrophobic interactions are identified as the driving forces for proteins binding to CDS-PMo_12_@PVP_0_ NPs, while the electrostatic interactions are identified as the main forces between proteins and CDS-PMo_12_@PVP_1_ NPs. The specific binding sites and contact surface area (CSA) were further visualized by molecular docking computational simulations. The CSA of proteins binding on CDS-PMo_12_@PVP_0_ NPs is larger than that of CDS-PMo_12_@PVP_1_ NPs. Importantly, the CSA of Cyt-C is larger than that of Hb and BSA on CDS-PMo_12_@PVP_x_(x = 0,1). Next, a series of CDS-PMo_12_@PVP_x_(x = 0 ~ 1) NPs were incubated with human plasma, the composition of the plasma protein corona was examined by label-free liquid chromatography mass spectrometry (LC–MS/MS). Along with the increase of PVP content, the number of identified protein groups covering the CDS-PMo_12_@PVP_x_(x = 0 ~ 1) NPs decreases gradually. Moreover, 76 differential adsorption proteins between CDS-PMo_12_@PVP_0_ and CDS-PMo_12_@PVP_1_ NPs are identified, in which apolipoprotein is up-regulated of the plasma corona proteins on CDS-PMo_12_@PVP_1_ NPs. Researches have proved that the adsorption of apolipoproteins can prolong circulation times [28]. Therefore, our studies demonstrate that PVP grafting on PMo_12_-based NPs mitigates plasma protein corona formation, which provides a new potential strategy for PMo_12_-based nanodrug design with better biological sustainability.

## Results and discussions

### Synthesis and characterization of CDS-PMo_12_@PVP_x_ (x = 0 ~ 1) NPs

**Fig. 1:**
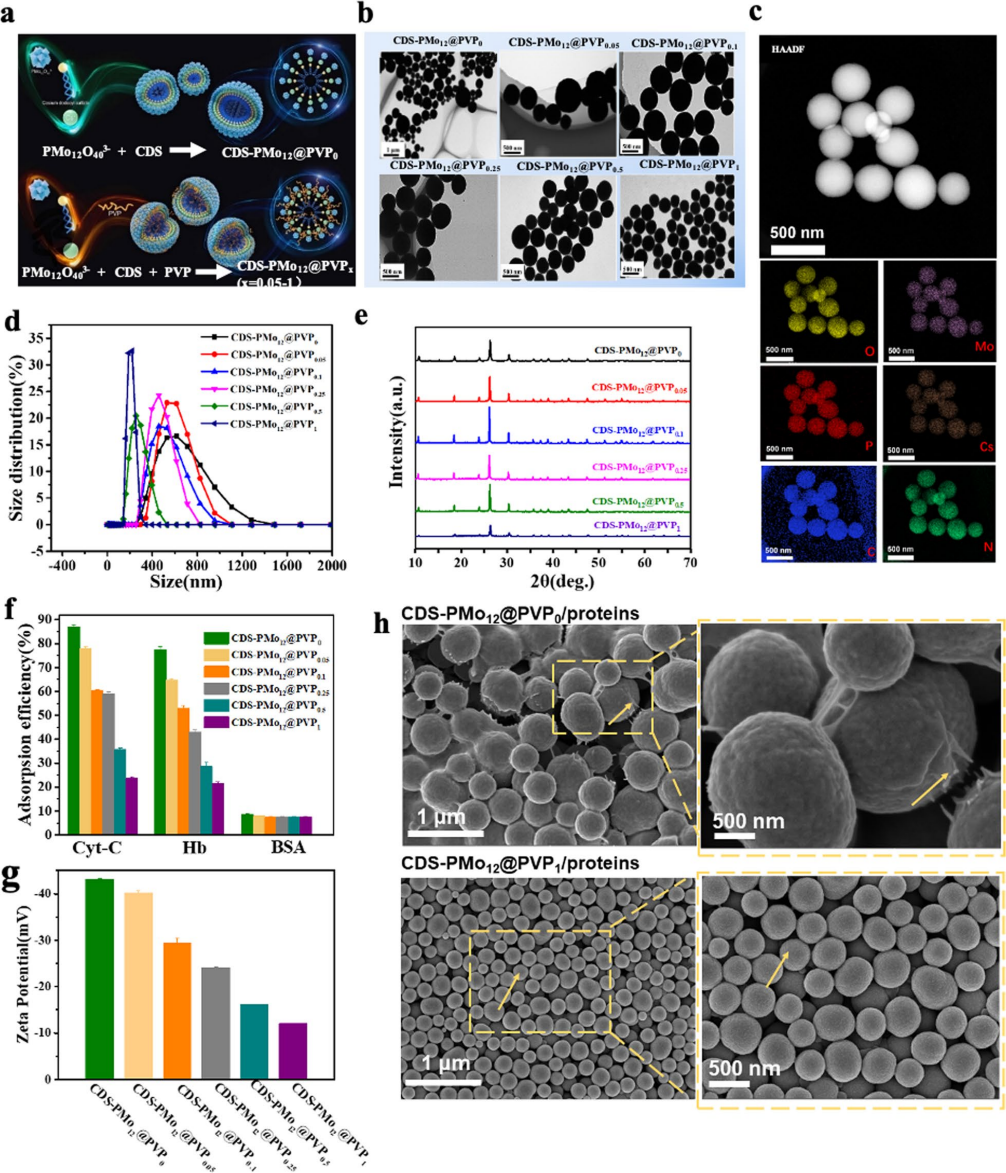
CDS-PMo_12_@PVP_x_(x = 0 ~ 1) NPs synthesis, characterization, and protein adsorption efficiency. **a** Schematic illustration of the formation process of CDS-PMo_12_@PVP_x_(x = 0 ~ 1) NPs. **b** TEM images of CDS-PMo_12_@PVP_x_(x = 0 ~ 1) NPs. **c** HAADF-STEM image of CDS-PMo_12_@PVP_1_ NPs and corresponding O, Mo, P, Cs, C, and N elemental mapping. **d** The hydrodynamic diameters (d_h_) and polydisperse index (PDI) values of CDS-PMo_12_@PVP_x_(x = 0 ~ 1) NPs characterized by dynamic light scattering (DLS). For CDS-PMo_12_@PVP_0_ (d_h_ = 613.8 ± 2.3, PDI = 0.197 ± 0.005), CDS-PMo_12_@PVP_0.05_ (d_h_ = 572.2 ± 2.7, PDI = 0.186 ± 0.01), CDS-PMo_12_@PVP_0.1_ (d_h_ = 496.7 ± 2.4, PDI = 0.181 ± 0.005), CDS-PMo_12_@PVP_0.25_ (d_h_ = 461.6 ± 4.1, PDI = 0.139 ± 0.007), CDS-PMo_12_@PVP_0.5_ (d_h_ = 310.1 ± 3.3, PDI = 0.128 ± 0.005), and CDS-PMo_12_@PVP_1_ (d_h_ = 240.1 ± 2.1, PDI = 0.119 ± 0.005), Standard deviations were calculated from three independent measurements. **e** XRD patterns of the CDS-PMo_12_@PVP_x_(x = 0 ~ 1) NPs. **f** The adsorption efficiency of CDS-PMo_12_@PVP_x_(x = 0 ~ 1) NPs towards three types of proteins. **g** the Zeta-potential of CDS-PMo_12_@PVP_x_(x = 0 ~ 1) NPs (B-R buffer, pH = 6). **h** SEM images of CDS-PMo_12_@PVP_0_ and CDS-PMo_12_@PVP_1_ NPs after absorbing the mixed solution of three types of proteins. Arrows indicate that the obvious protein coating induce rough surfaces of CDS-PMo_12_@PVP_0_ NPs and very few proteins coating on the surface of CDS-PMo_12_@PVP_1_ NPs

The CDS-PMo_12_@PVP_x_(x = 0 ~ 1) NPs were prepared by a micelle-based approach (Fig. 1a) with detailed experiment process given in the Additional file 1: Fig. 1b and Additional file 1: Fig. S1 provide the transmission electron microscopy (TEM) images of CDS-PMo_12_@PVP_x_(x = 0 ~ 1) NPs. CDS-PMo_12_@PVP_0_ NPs are heterogeneous, with sizes ranging from 100 to 1000 nm. The CDS-PMo_12_@PVP_x_(x = 0 ~ 1) NPs size distributions are statistically analyzed from TEM images by ImageJ software (Additional file 1: Fig. S2). The main size of CDS-PMo_12_@PVP_x_(x = 0.05 ~ 1) NPs are approximately 520, 482, 454, 300, 235 nm, respectively. The size of particles became smaller (from 520 to 235 nm) and more homogeneous with the increase of PVP content. Besides, the morphology of CDS-PMo_12_@PVP_1_ NPs were characterized by the high-angle annular darkfield scanning transmission electron microscopy (HAADF-STEM, Fig. 1c), and the corresponding elemental compositions were analyzed by energy-dispersive X-ray spectroscopy (EDS) in HAADF-STEM. According to the HAADF-STEM-EDS elemental mapping, we found that the O, Mo, P, Cs, C, and N elements exist simultaneously, illustrating the PVP successfully anchored to the CDS-PMo_12_@PVP_1_ NPs [25]. The hydrodynamic diameters (d_h_) and polydisperse index (PDI) values of CDS-PMo_12_@PVP_x_(x = 0 ~ 1) NPs was characterized by dynamic light scattering (DLS) (Fig. 1d) and the corresponding values are presented in the caption of Fig. 1d. Figure 1e showed the X-ray diffraction (XRD) patterns of CDS-PMo_12_@PVP_x_(x = 0 ~ 1) NPs. The 2θ diffraction peaks correspond to the crystalline phase peaks of the H_3_[PMo_12_O_40_] [29], indicating that the prepared PMo_12_-based NPs retain the H_3_[PMo_12_O_40_] Keggin structure. The peak intensity of XRD is mainly related to the crystallinity of the crystal. Therefore, the higher the degree of crystallization, the higher the intensity of XRD peak. When the content of PVP (0.1 ~ 1) in the reaction system increases, the order structure of crystals decreases, resulting in poor crystallinity, so the intensity of XRD becomes lower. On the other hand, it has been confirmed that the peak intensity of XRD is also influenced by the nanoparticles’ size, the smaller size of nanoparticles, and the higher intensity of XRD the peak. This explains why the XRD increases with the increasing PVP content (0 ~ 0.1). The FT-IR spectra of CDS-PMo_12_@PVP_0_, CDS-PMo_12_@PVP_0.5_, and CDS-PMo_12_@PVP_1_ showed the presence of absorption bands of the PMo_12_O_40_^3−^ (P-O_a_ at 1064 cm^−1^, Mo-O_d_ at 962 cm^−1^, Mo-O_b_ at 869 cm^−1^, and Mo-O_c_ at 790 cm^−1^) in the fingerprint area (Additional file 1: Fig. S3) [24], which indicates that the PMo_12_O_40_^3−^ in the nanocomposites remain the Keggin structure. The absorption bands of CDS-PMo_12_@PVP_0.5_ and CDS-PMo_12_@PVP_1_ arising from the PMo_12_O_40_^3−^ are shifted in position, which demonstrates that the bonds of the PMo_12_O_40_^3−^ are either strengthened or weakened, owing to the interaction between the N of the PVP and O atom of the PMo_12_O_40_^3−^. Besides, the resonance peak of C–O (at 1643 cm^−1^) shows no change, and the peak of the N–OH complex (at 1288 cm^−1^) disappears as compared with the PVP spectrum. These changes in the spectrum of CDS-PMo_12_@PVP_1_ suggest the coordination between N and PMo_12_O_40_^3−^ as the main reaction, while the reaction between O and PMo_12_O_40_^3−^ is less significant. Overall, these results demonstrate PVP was successfully anchored to the PMo_12_-based NPs.

### Protein adsorption behaviors on the CDS-PMo_12_@PVP_x_(x = 0 ~ 1) NPs

Cyt-C, Hb, and BSA were chosen as representatives of basic, neutral, and acidic proteins to evaluate adsorption behaviors of the CDS-PMo_12_@PVP_x_(x = 0 ~ 1) NPs. Additional file 1: Fig. S4 summarized the protein adsorption performance on CDS-PMo_12_@PVP_0_ NPs. The optimized adsorption conditions from preliminary experiments (adsorption time: 20 min, temperature: 25 °C, B-R buffer concentration: 0.04 mol L^−1^) were applied to the adsorption study of CDS-PMo_12_@PVP_x_(x = 0 ~ 1) NPs [30]. The adsorption efficiencies of Hb and Cyt-C (Hb/Cyt-C) on CDS-PMo_12_@PVP_x_(x = 0 ~ 1) NPs were gradually decreased when PVP content increased, while no obvious alteration in the adsorption efficiencies of BSA was observed (Fig. 1f). Zeta-potential measurements illustrate that the neutral amphiphilic PVP polymer neutralizes the electronegativity of the CDS-PMo_12_@PVP_0_ NPs (Fig. 1g), limiting the adsorption of positively charged Hb/Cyt-C proteins on the CDS-PMo_12_@PVP_x_(x = 0.05 ~ 1) NPs at pH 6, hence explains the declined adsorption efficiencies. On the other hand, BSA (pI = 4.7) is negatively charged at pH 6, and virtually no retention of BSA occurs on the negatively charged CDS-PMo_12_@PVP_0_ NPs surfaces. The SEM images show obvious protein coatings on the surface of CDS-PMo_12_@PVP_0_ NPs and barely any retention on the surface of CDS-PMo_12_@PVP_1_ NPs (Fig. 1h) after absorbing the mixed solution of three types of proteins. Our results confirm that PVP in the nanocomposites regulates the chemical composition, particle size, surface physicochemical property, and consequently, protein adsorption efficiency.

### The binding process and mechanism of protein adsorption on PMo_12_-based NPs

**Fig. 2:**
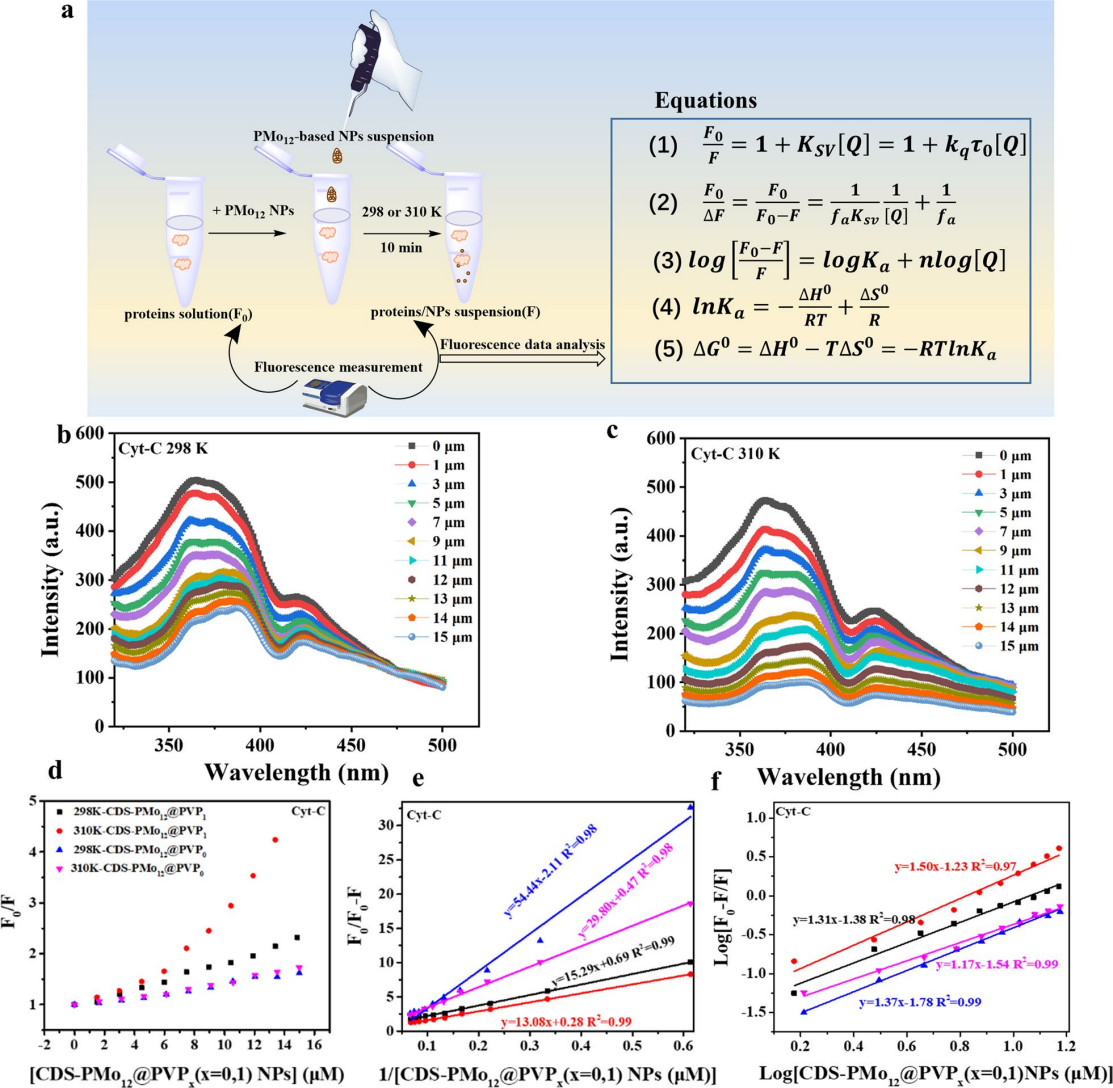
The fluorescence quenching studies of proteins in the presence of CDS-PMo_12_@PVP_x_(x = 0,1) NPs. **a** The fluorescence quenching experiment and corresponding calculation equation. **b**, **c** The fluorescence emission spectra of Cyt-C in the absence and presence of CDS-PMo_12_@PVP_0_ NPs at 298 K and 310 K, respectively. The concentration of Cyt-C is fixed as 5 μM; the concentrations of CDS-PMo_12_@PVP_0_ NPs is changed from 0 to 15 μM. **d** Stern–Volmer plot of Cyt-C intrinsic fluorescence quenching by CDS-PMo_12_@PVP_x_(x = 0,1) NPs. **e** The modified Stern–Volmer plot of Cyt-C intrinsic fluorescence quenching by CDS-PMo_12_@PVP_x_(x = 0,1) NPs. **f** The double logarithmic plot of Cyt-C intrinsic fluorescence quenching by CDS-PMo_12_@PVP_x_(x = 0,1) NPs at 298 K and 310 K, respectively

To explore the effect of PVP on the binding kinetics and thermodynamic process of protein/PMo_12_-based NPs complexes formation, we investigated the protein fluorescence quenching process upon binding to (CDS-PMo_12_@PVP_x_(x = 0,1) NPs quenchers [31]. First, we incubated proteins with different concentrations of CDS-PMo_12_@PVP_x_(x = 0,1) NPs ranging from 0 to 15 μM at 298 K and 310 K for 10 min, respectively. Then we measured the intrinsic fluorescence intensity (here tryptophan residue of Cyt-C, Hb, and BSA proteins) before and after incubation with CDS-PMo_12_@PVP_x_(x = 0,1) NPs and analyzed fluorescence quenching data by the Stern–Volmer (S–V) equation (Eq. 1) [32].

1 $$\frac{{F}_{0}}{F}=1+{K}_{SV}\left[Q\right]=1+{k}_{q}{\tau }_{0}\left[Q\right],$$

where F_0_ and F are the fluorescence intensities of proteins in the absence and presence of quencher (here CDS-PMo_12_@PVP_x_(x = 0,1) NPs); *K*_*SV*_ is the S-V quenching constant; [Q] is the total concentration of the quencher; *k*_*q*_ is the quenching rate constant, and τ_0_ is the fluorophore average lifetime in the absence of quencher (for biomolecules is 10^−8^ s). The fluorescence spectra of proteins incubating with CDS-PMo_12_@PVP_x_(x = 0,1) and S-V plot are displayed in Fig. 2b–d and Additional file 1: Figs. S5–S7. The S-V plot of proteins binding to CDS-PMo_12_@PVP_x_(x = 0,1) NPs shows a positive deviation from a linear S-V relation (Fig. 2d and Additional file 1: Fig. S7). The positive deviation of the slope is attributed to the simultaneous presence of dynamic and static quenching [33–37].

A modified S-V equation (Eq. 2) was used to calculate the effective S-V quenching constant (*K*_sv_) and the quenching rate constant (*k*_q_) of the interaction of proteins and CDS-PMo_12_@PVP_x_(x = 0,1) NPs.

2 $$\frac{{F}_{0}}{\Delta F}=\frac{{F}_{0}}{{F}_{0}-F}=\frac{1}{{f}_{a}{K}_{sv}}\frac{1}{\left[Q\right]}+\frac{1}{{f}_{a}}.$$

In which *f*_a_ is the mole fraction of accessible fluorescence, and *K*_*sv*_ is the effective S-V quenching constant for the accessible fluorophores. The dependence of F_0_/(F_0_-F) vs [*Q*]^−1^, should be linear with the slope of (*faKsv*)^−1^, whereas the value *fa*^−1^ is fixed on the ordinate. Therefore, the effective quenching constant *Ksv* is a quotient of the ordinate *fa*^−1^ and the slope (*faKsv*)^−1^. The modified S–V plot of proteins binding to CDS-PMo_12_@PVP_x_(x = 0,1) NPs are shown in Fig. 2e and Additional file 1: Fig. S7. The values of *K*_*sv*_ for BSA/CDS-PMo_12_@PVP_0_, Hb/CDS-PMo_12_@PVP_0_, Cyt-C/CDS-PMo_12_@PVP_1_, and Hb/CDS-PMo_12_@PVP_1_ complexes are increased with the temperature (Table 1), indicating a dynamic process exists in those complexes. The *Ksv* of Cyt-C/CDS-PMo_12_@PVP_0_ and BSA/CDS-PMo_12_@PVP_1_ complexes are correlated inversely with temperature, indicating the proteins quenching mechanism is initiated by static quenching. However, the *Kq* of the proteins/CDS-PMo_12_@PVP_x_(x = 0,1) complexes are higher than the maximal dynamic quenching constant (2 × 10^10^ M^−1^ s^−1^), revealing the main quenching mechanism for proteins binding to CDS-PMo_12_@PVP_x_(x = 0,1) NPs is static quenching [38].

**Table 1:** The adsorption kinetics and thermodynamic parameters of the protein/PMo12-based NPs complexes obtained from fluorescence quenching data

Proteins/PMo_12_ NPs	Temp (K)	*K*sv	*K*q (×10^12^)	fa	n	*K*_a_ (×10^6^M^-1^)	*Δ*G^0^ (kJ/mol)	*Δ*H^0^ (kJ/mol)	*Δ*S^0^ (J/mol^.^ K)
**BSA/CDS-PMo**_**12**_**@PVP**_**0**_	**298**	**0.085**	**8.46**	**0.476**	**0.94**	**0.035**	**−** **25.97**	**144.47**	**571.66**
	**310**	**0.597**	**59.7**	**0.625**	**0.54**	**0.338**	**−** **32.83**		
**Hb/CDS-PMo**_**12**_**@PVP**_**0**_	**298**	**0.013**	**1.4**	**2.381**	**1.2**	**0.028**	**−** **25.46**	**19.01**	**149.17**
	**310**	**0.039**	**3.9**	**1.099**	**1.1**	**0.038**	**−** **27.31**		
**Cyt-C/CDS-PMo**_**12**_**@PVP**_**0**_	**298**	**0.045**	**4.52**	**1.449**	**1.3**	**0.04**	**−** **26.31**	**22.14**	**162.5**
	**310**	**0.021**	**2.14**	**3.571**	**1.5**	**0.06**	**−** **28.26**		
**BSA/CDS-PMo**_**12**_**@PVP**_**1**_	**298**	**0.064**	**6.42**	**0.303**	**0.78**	**0.016**	**−** **24.08**	**−** **8.93**	**50.83**
	**310**	**0.005**	**0.531**	**3.333**	**0.71**	**0.014**	**−** **24.69**		
**Hb/CDS-PMo**_**12**_**@PVP**_**1**_	**298**	**0.012**	**1.2**	**2.439**	**0.91**	**0.019**	**−** **24.48**	**−** **2.87**	**72.48**
	**310**	**0.032**	**3.3**	**0.971**	**0.83**	**0.018**	**−** **25.35**		
**Cyt-C/CDS-PMo**_**12**_**@PVP**_**1**_	**298**	**0.004**	**4.04**	**4.545**	**0.98**	**0.032**	**−** **25.74**	**−** **7.35**	**61.67**
	**310**	**0.016**	**1.58**	**2.128**	**1.1**	**0.028**	**−** **26.48**		

The number of binding sites (*n*)*,* and binding constant (*K*_a_) were obtained according to the double logarithmic equation (Eq. 3) [39].

3 $$log\left[\frac{{F}_{0}-F}{F}\right]=log{K}_{a}+nlog\left[Q\right].$$

The double logarithmic plot of proteins binding to CDS-PMo_12_@PVP_x_(x = 0,1) NPs are shown in Fig. 2f and Additional file 1: Fig. S7. By linear fitting for the the double logarithmic plot, the values of *n* and *K*_a_ are obtained from the slope and Y-axis intercept, respectively. The value of *n* for Cyt-C/Hb binding to CDS-PMo_12_@PVP_1_ NPs is smaller than that binding to CDS-PMo_12_@PVP_0_ NPs (Table 1). The results suggest that the introduction of PVP reduces the number of binding sites of proteins on CDS-PMo_12_@PVP_1_ NPs. Since the values of *K*_*a*_ for Cyt-C/Hb binding to CDS-PMo_12_@PVP_0_ NPs have no apparent increase with temperature (Table 1), combined with the static quenching mechanism, we cross-verified the stable complex formation between Cyt-C/Hb and CDS-PMo_12_@PVP_0_ NPs. Conversely, with the temperature rising, the value of *K*_*a*_ increases largely for BSA/CDS-PMo_12_@PVP_0_ complex (Table 1), suggesting the BSA/CDS-PMo_12_@PVP_0_ complex is unstable [40–42]. *K*_a_ is dependent on temperature, which indicates that the protein formation on CDS-PMo_12_@PVP_x_(x = 0,1) NPs is a thermodynamic process. Enthalpy change (*∆H*^0^), entropy change (*∆S*^0^), and free energy change (*ΔG*^*0*^) are used to further characterize the driving interaction force between CDS-PMo_12_@PVP_x_(x = 0,1) NPs and three types of proteins. The values of *∆S*^*0*^ and *∆H*^*0*^ were calculated from the slope and the intercept, respectively, by fitting linearly to the plot of ln*K*_*a*_ Vs. 1/T according to the Van Hoff equation Eq. (4), whereas the *ΔG*^*0*^ value was calculated from Eq. (5).

4 $$ln{K}_{a}=-\frac{\Delta {H}^{0}}{RT}+\frac{\Delta {S}^{0}}{R},$$

where *K*_a_ is the binding constant at the corresponding temperature (*T*) and *R* is the gas constant. *∆S*^0^ and *∆H*^0^ are determined from the linear Van’t Hoff plots. *ΔG*^*0*^ is estimated from the following equation (Eq. 5) [43]:

5 $$\Delta {G}^{0}=\Delta {H}^{0}-T\Delta {S}^{0}=-RTln{K}_{a}.$$

According to the views of Timasheff [44], the positive values of *∆S*^*0*^ and *∆H*^*0*^ indicate that hydrophobic forces plays a major role in protein interaction with CDS-PMo_12_@PVP_0_ NPs (Table 1). A negative value of *∆H*^*0*^ and positive values of *∆S*^*0*^ indicate that electrostatic forces are the major forces between the CDS-PMo_12_@PVP_1_ and proteins (Table 1). The negative sign of the *ΔG*^*0*^ proves that the CDS-PMo_12_@PVP_x_(x = 0,1) NPs interact with three types of proteins are spontaneous (Table 1).

### Molecular docking study of CDS-PMo_12_@PVP_x_(x = 0,1) NPs and protein interactions

**Fig. 3:**
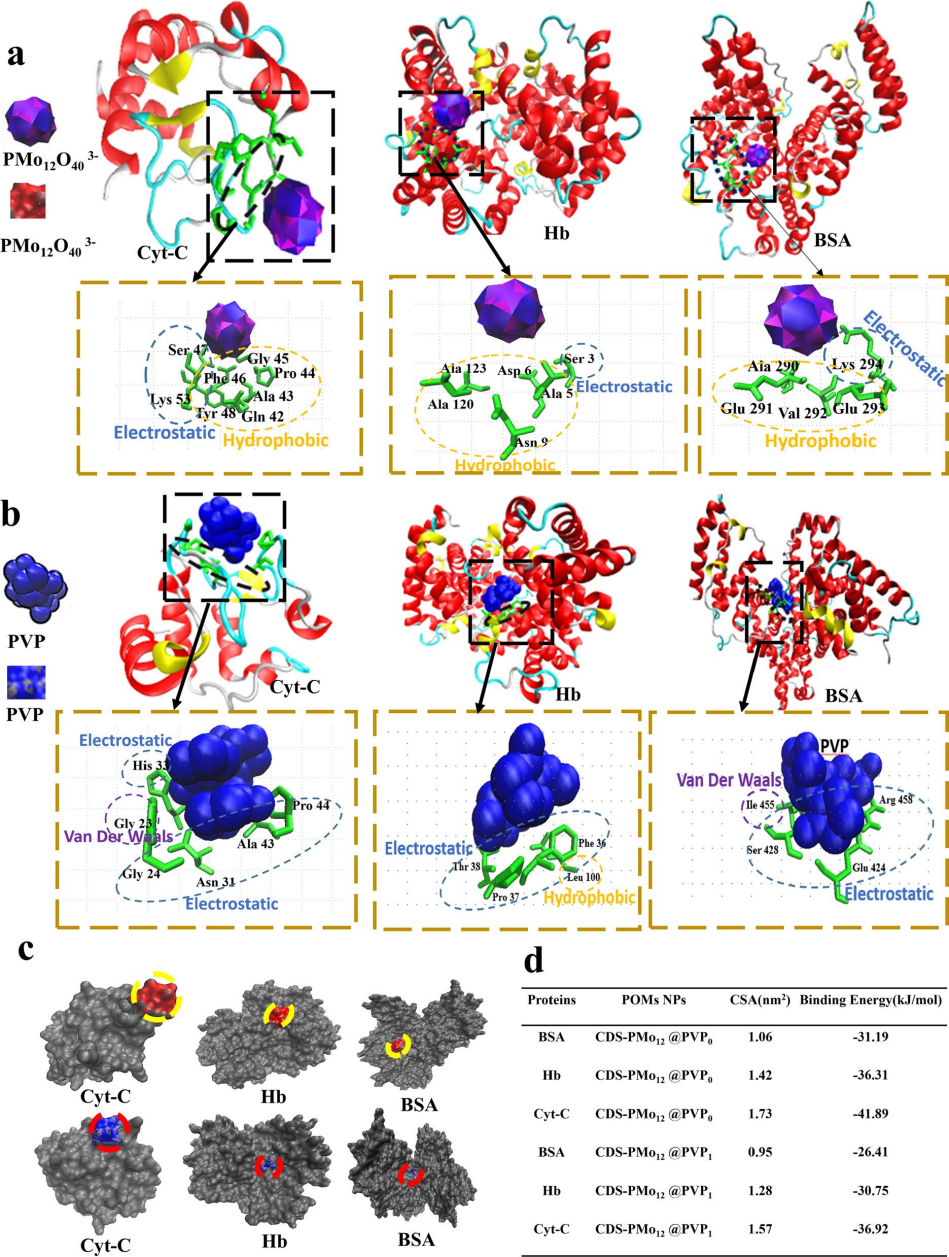
The molecular docking computational results of the interaction of Cyt-C, Hb, and BSA with CDS-PMo_12_@PVP_x_(x = 0,1) NPs. The structure snapshots of Cyt-C, Hb, and BSA adsorption on (**a**) CDS-PMo_12_@PVP_0_ and (**b**) CDS-PMo_12_@PVP_1_ NPs, respectively. **c** The CSA visualization of the three model proteins binding to the CDS-PMo_12_@PVP_0_ NPs (the upper row) and binding to CDS-PMo_12_@PVP_1_ NPs (the lower row). **d** The value of calculated CSA and the binding energy of three model proteins on CDS-PMo_12_@PVP_x_ (x = 0,1) NPs

Molecular docking was further performed on the interaction of PMo_12_-based NPs with proteins to identify the binding orientation of CDS-PMo_12_@PVP_0_ and CDS-PMo_12_@PVP_1_ onto proteins. The docking results indicate that hydrophobic forces is the main driving forces for CDS-PMo_12_@PVP_0_ binding to three types of proteins, and while the electrostatic interactions are identified as the main forces between proteins and CDS-PMo_12_@PVP_1_ NPs. During CDS-PMo_12_@PVP_0_ interacts with the proteins, the primary force is hydrophobic and the secondary forces is electrostatic (Fig. 3a). The contact surface area (CSA) between the model proteins and the PMo_12_-based NPs was calculated using molecular docking. Molecular docking [45] results show that CDS-PMo_12_@PVP_1_ binds with Cyt-C, Hb, and BSA mainly through electrostatic interaction, but also Van der Waals force as the second action force (Fig. 3b).

The contact surface area (CSA) and binding energy between the three model proteins and CDS-PMo_12_@PVP_x_(x = 0,1) NPs were calculated using molecular docking [45]. As shown in Fig. 3c, d, the CSA of proteins on CDS-PMo_12_@PVP_0_ is larger than that on CDS-PMo_12_@PVP_1_. Importantly, the CSA of Cyt-C is larger than that of Hb/BSA on CDS-PMo_12_@PVP_x_(x = 0,1) NPs. The binding energy of the three model proteins binding to CDS-PMo_12_@PVP_0_ is smaller than that binding to CDS-PMo_12_@PVP_1_, thus confirming a higher affinity of three model proteins to CDS-PMo_12_@PVP_0_ NPs than CDS-PMo_12_@PVP_1_ NPs. Thus, the lower binding affinity of proteins to CDS-PMo_12_@PVP_1_ NPs mitigate the protein adsorption. It is generally believed that reducing biofouling could significantly attenuate subsequent adverse inflammatory responses including leukocyte activation, tissue fibrosis, thrombosis coagulation, and infection [46].

### Comparison of CDS-PMo_12_@PVP_x_(x = 0 ~ 1) NPs in human plasma protein corona formation

**Fig. 4:**
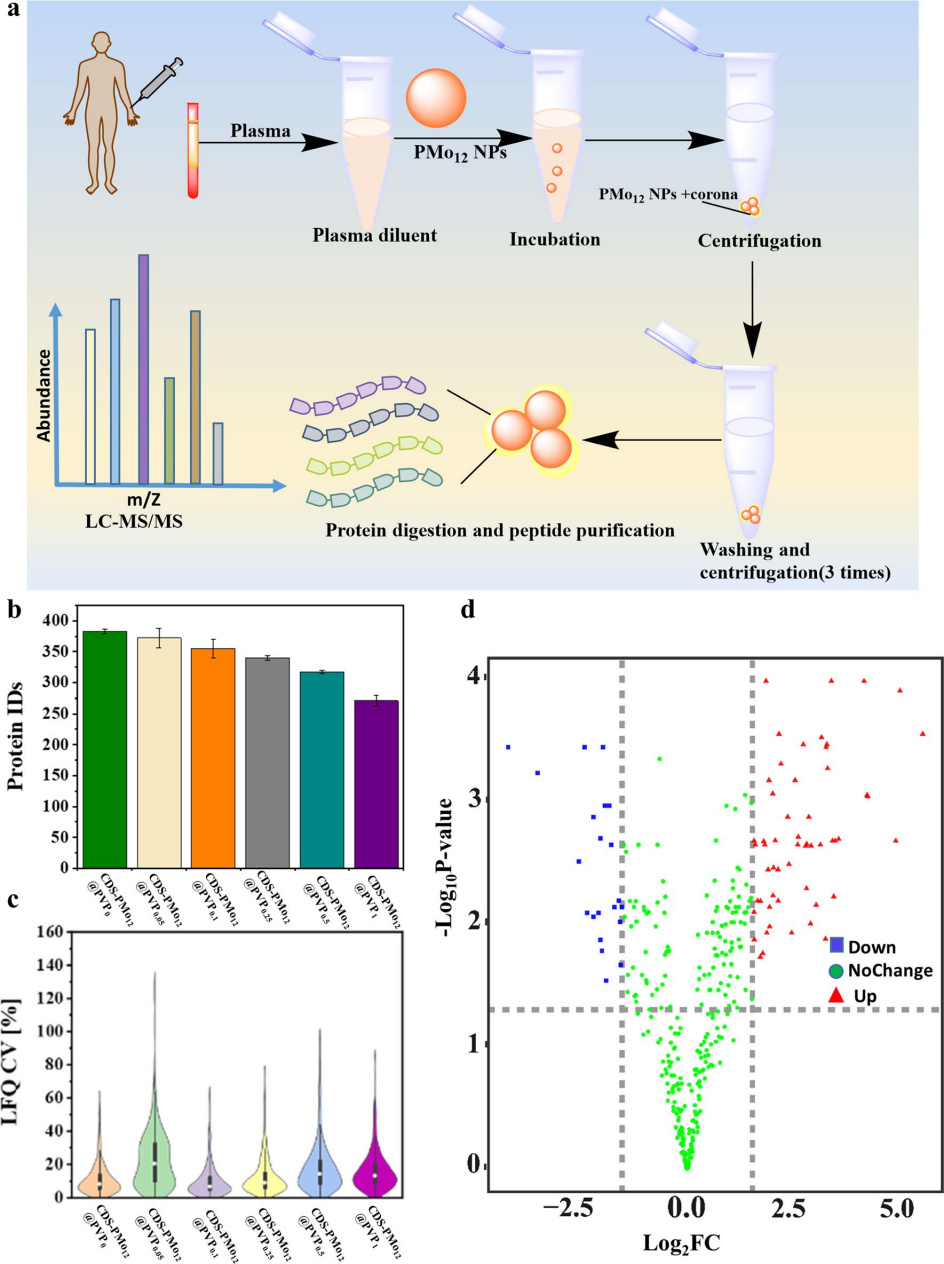
Plasma Proteomics characterization of the CDS-PMo_12_@PVP_x_(x = 0 ~ 1) NPs. **a** The plasma protein corona formation process of CDS-PMo_12_@PVP_x_(x = 0 ~ 1) NPs, and Plasma Proteomics identified by LC–MS/MS. **b** The number of plasma protein groups identified by LC–MS/MS from the protein corona of the CDS-PMo_12_@PVP_x_(x = 0 ~ 1) NPs, standard deviation across three assay replicates are shown as error bar. **c** CV% for precision evaluation of three assay replicates. Inner boxplots report the 25% (lower hinge), 50%, and 75% quantiles (upper hinge). Whiskers indicate observations outside hinge ± 1.5* interquartile range (IQR). Violin plots capture all data points. **d** Volcano plot of the correlations between the P-value and fold change (FC), the normalized abundance ratio of CDS-PMo_12_@PVP_1_/CDS-PMo_12_@PVP_0_ was used for the calculation the FC.(number of parallel = 3, P-values were calculated with Student’s t-test)

The CDS-PMo_12_@PVP_x_(x = 0 ~ 1) NPs were then incubated in human plasma to acquire a stable protein corona, followed by centrifugation of NPs from unbound proteins. The plasma proteins in the protein corona were then digested, purified, and eluted. The resulting peptides from the NPs-bound corona were analyzed by LC–MS/MS coupled with label-free quantification in data-dependent acquisition mode (DDA) [47] (Fig. 4a, see details in “Experimental and methods”).

CDS-PMo_12_@PVP_0_, CDS-PMo_12_@PVP_0.05_, CDS-PMo_12_@PVP_0.1_, CDS-PMo_12_@PVP_0.25_, CDS-PMo_12_@PVP_0.5_, and CDS-PMo_12_@PVP_1_ NPs facilitated the quantification of ~ 300 protein groups across 18 samples (triplicate measurements of the six NPs) (Fig. 4b). Compared to the protein groups on CDS-PMo_12_@PVP_0_ (383), those on CDS-PMo_12_@PVP_0.05_, CDS-PMo_12_@PVP_0.1_, CDS-PMo_12_@PVP_0.25_, CDS-PMo_12_@PVP_0.5_, and CDS-PMo_12_@PVP_1_ decreased to 372, 355, 340, 317, and 271, respectively (Fig. 4b), further supporting that the PVP addition indeed quantitatively decreases the protein adsorption. To determine the triplicate tests variability, we further calculated the coefficient of variation (CV) for protein group quantification using DDA. The coefficient of variation (CV) of triplicate tests were 9.64%, 21.3%, 7.74%, 10.32%, 15.48% and 14.35% (on average 13.14%) for CDS-PMo_12_@PVP_0_, CDS-PMo_12_@PVP_0.05_, CDS-PMo_12_@PVP_0.1_, CDS-PMo_12_@PVP_0.25_, CDS-PMo_12_@PVP_0.5_, and CDS-PMo_12_@PVP_1_, respectively (Fig. 4c). The number of protein groups with CVs < 20% agrees with the range of the precision determined in previous studies [48, 49]. To explore how the PVP influences the plasma protein corona of the PMo_12_-based NPs, we further compared the plasma protein corona compositions formed on the CDS-PMo_12_@PVP_0_ and CDS-PMo_12_@PVP_1_ NPs by statistical analysis. Compared to CDS-PMo_12_@PVP_0_ protein corona compositions, a total of 76 significant differentially proteins (with adjusted P-value < 0.01) are identified for CDS-PMo_12_@PVP_1_ protein corona composition, among which 55 proteins are up-regulated and 21 proteins are down-regulated (Fig. 4d). The relative abundance of differential proteins is shown in the heatmap (Additional file 1: Fig. S7). The top ten up-regulated proteins and down-regulated proteins are listed in Additional file 1: Table S1. Apolipoprotein, Plasma protease C1 inhibitor, and IgG Fc-binding protein OS = Homo sapiens are significantly up-regulated on CDS-PMo_12_@PVP_1_ protein corona, compared to the compositions of CDS-PMo_12_@PVP_0_ protein corona. As the molecular identification of the human corona suggests, apolipoprotein is the second most abundant class of proteins, proposed to have dysoponic activity (i.e. favor long blood circulation) [28]. LC/MS–MS analysis results further verified that PVP grafting on the PMo_12_-based NPs successfully mitigates the plasma protein corona formation and apolipoproteins adsorption on CDS-PMo_12_@PVP_1_ NPs could potentially extend blood circulation time and permit better bio-sustainability.

## Conclusions

The PMo_12_-based NPs are reported to deliver promising anti-tumor biological activities by the virtue of their desired diversity in structures and properties. However, nanomaterials’ effective nanomedicine applications are hampered by limited understanding and control over their interactions with complex biological systems. Here, we adopted PVP polymer to modulate the size and surface functionality of PMo_12_-based NPs. PVP successfully decreased the protein adsorption on the surface of CDS-PMo_12_@PVP_x_(x = 0 ~ 1) NPs. On the assessment of the interaction mechanism between engineering PMo_12_-based NPs and proteins (base, neutral and acid proteins), the steady-state fluorescence quenching results revealed the interaction between model proteins and CDS-PMo_12_@PVP_0_ NPs occurs spontaneously mainly by hydrophobic forces, whereas the electrostatic interactions make the main forces between proteins and CDS-PMo_12_@PVP_1_ NPs. Molecular docking results indicated that the introduction of PVP reduces the number of binding sites and contact surface area. LC–MS/MS further indicated that the PVP reduces the plasma proteins adsorption on the CDS-PMo_12_@PVP_1_ NPs. In addition, apolipoprotein as the main composition of adsorption proteins on CDS-PMo_12_@PVP_1_ NPs is proposed to have dysoponic activity, enhancing the circulation time. Overall, the surface physicochemical properties of NPs have a significant impact on the adsorption of proteins. We believe such regulation of the surface physicochemical of NPs and in-depth understanding of the protein adsorption process can effectively facilitate the design of PMo_12_-based nanodrug.

## Experimental and methods

### Reagents

Keggin-type phosphomolybdic acid H_3_PMo_12_O_40_^.^xH_2_O (PMo_12_), sodium dodecyl sulfate (SDS), cesium carbonate (Cs_2_CO_3_), Polyvinylpyrrolidone (PVP K30, average Mw: 300 000) were obtained from Alfa Aesar. Cyt-C (30398, 95%), Hb (H2625, 95%), BSA (A3311, 95%) proteins, and ammonium hydrogen carbonate (NH_4_HCO_3_) were purchased from Sigma-Aldrich (St. Louis, MO, USA) and used without further purification. Sodium citrate, citric acid, acetonitrile, isopropyl alcohol, acetone, ethyl alcohol, and guanidine hydrochloride were acquired from Sinopharm Chemical Reagent (Shanghai, China). Radio immunoprecipitation assay lysis buffer (RIPA) was purchased form Beyotime technology. Macro spin column TARGA C18 was purchased from The Nest Group, Protein LoBind tubes and ultrafiltration tubes (10 kDa) were purchased from Eppendorf. DL-dithiothreitol (DTT) and iodoacetamide (IAA) were supplied by Acros Organics. Sequencing grade modified trypsin (Promega), formic acid (FA), acetonitrile (ACN), methanol, acetic acid, and Pierce BCA protein assay kit were purchased from Thermo Fisher Scientific. Deionized water (ρ = 18 MΏ cm, 25 °C) was obtained from a Millipore Milli-Q water purification system.

### Fabrication and characterization of the CDS-PMo_12_@PVP_x_(x = 0 ~ 1) NPs

CDS-PMo_12_@PVP_x_(x = 0 ~ 1) NPs were synthesized according to the previously described approach with some modifications [25]. Detailed procedures are given in the experimental part of the SI. The morphology of the CDS-PMo_12_@PVP_x_(x = 0 ~ 1) NPs was observed with transmission electron microscopy (biology TEM, Tecnai G2 spirit Biotwin operated at 120 kV; Talos F200X G2 operated at 200 kV). The crystalline structure was recorded by X-ray diffractometer (XRD) (Bruker AXS D8 Focus), using Cu Ka radiation (λ = 1.54056 A). Fourier transform infrared (FT-IR) spectra were acquired from a Nicolet 50 FT-IR spectrometer (Thermo Fisher Scientific, Waltham, MA, USA), ranging from 400 to 4000 cm^−1^. The UV–Vis absorption spectra were measured on UV 2600 UV–Vis spectrophotometer. The nanoparticles distribution characteristics and zeta-potential were measured using a particle size analyzer (Malvern, Nano ZS, Japan). Fluorescence measurements were performed on a Jobin Yvon Horiba Fluoromax-3 spectrophotometer.

### Protein adsorption behavior on the CDS-PMo_12_@PVP_x_(x = 0 ~ 1) NPs

BSA, Hb, and Cyt-C were chosen as models of acidic, neutral, and basic proteins to evaluate the proteins’ adsorption behavior on the CDS-PMo_12_@PVP_x_(x = 0 ~ 1) NPs. The experiment procedure is below: 1.0 mL of protein solution was mixed with 5.0 mg of CDS-PMo_12_@PVP_x_(x = 0 ~ 1) NPs and the mixture was shaken vigorously for 20 min to facilitate the adsorption of proteins. The pH of the protein solution was controlled by Britton-Robinson (B-R) buffer (a mixture of 40 mmol/L phosphoric acid, acetic acid, boric acid, and adjusted by 200 mmol/L sodium hydroxide) within a range of 3–7. After the adsorption process, the solid and liquid phase was separated by centrifugation at 8000 rpm for 6 min and the residual proteins in the aqueous phase were monitored using a UV–vis spectrophotometer in a 1.0 cm quartz cell by measuring the characteristic adsorption at 406 nm for Hb, 409 nm for Cyt-C, and 595 nm for BSA directly. The protein adsorption efficiency was calculated based on the protein concentration before and after adsorption.

### Steady-state fluorescence quenching measurements

The fluorescence emission spectra of BSA, Hb, and Cyt-C were measured at a constant concentration (5 μM) in the presence of an increasing concentration of PMo_12_-based NPs (0–15 µM in particles). A certain concentration of PMo_12_-based NPs was added to the protein solutions and incubated for 10 min, and then the mixture was transferred to a quartz cuvette, and their fluorescence spectra were acquired in the range of 300–600 nm when excited at 290 nm. The area under each fluorescence curve was integrated and used to measure the free BSA concentration using a standard calibration curve. The cuvette path length for fluorescence quenching measurements was 1 cm. The slit widths of excitation and emission were set both at 10 nm, respectively.

### Molecular docking

The molecular docking is done on AutoDock Vina software [50] to predict binding parameters, the contact surface area (CSA), and binding energy of three model proteins (BSA, Hb, and Cyt-C) with target nanoparticles. The crystal structures of three model proteins (BSA: 4F5S [51], Hb: 1G09 [52], and Cyt-C: 2B4Z [53]) used in molecular docking studies were obtained from the RCSB Protein Data Bank (http://www.rcsb.org). The rigid docking method was utilized to evaluate all possible binding sites on three model proteins. The outer surface of target nanoparticles as the interaction area was considered for molecular docking calculation. So, the H_3_PMo_12_O_40_ and PVP molecular structures were built using the Hyperchem 8.0.6 program. The geometry of PMo_12_O_40_^3−^ polyanion and PVP polymer were optimized to minimal energy employing the theoretical level of B3LYP with LanL2DZ (for Mo atom) and 6-31G (for P, O, N, C, and H atoms) basis sets and implemented in Gaussian 98 program. The AutoDock Vina instruction was considered for input file preparation. The PDBQT format of three model proteins, PMo_12_O_40_^3−^ polyanion [54], and PVP input files were prepared using the AutoDock Tools 1.5.4 package [50]. Each molecular docking calculation produced 20 binding mode states poses with the exhaustiveness parameter value equal to 1000. In all cases, the binding energy and root-mean-square deviation (RMSD) are considered together and the one with the best affinity is selected as the optimal binding mode. After selecting the best mode of interaction, the CSA was calculated by the related code in VMD package [55] was used to analyze molecular docking results. It is worth mentioning that we repeated the docking calculations three times and obtained the same values.

### Protein corona preparation and proteomic analysis

Plasma samples were diluted 1:5 in B-R buffer (a mixture of 40 mmol/L phosphoric acid, acetic acid, boric acid, and adjusted by 200 mmol/L sodium hydroxide). To form the protein corona, 0.5 g CDS-PMo_12_@PVP_x_(x = 0 ~ 1) NPs was mixed with 500 μL diluted plasma samples in a tube. The tube was sealed and incubated at 37 °C for 1 h with shaking at 300 rcf. After incubation, the mixture was centrifuged to separate the nanoparticle-protein complexes from plasma solution for at least 20 min at 4 °C at 15,000 rcf. Discard the supernatant and wash the pellet with ddH_2_O (300 μL). The protein corona was further washed with 200 μL of ddH_2_O three times with centrifugal separation. Elute the proteins from the nanoparticles by adding 100 μL of RIPA lysis buffer and incubate for 5 min at 95 °C. Pellet the nanoparticles by centrifugation for 15 min at 15,000 rcf at room temperature, and then transfer the supernatant containing eluted corona proteins to a fresh tube. Determine the protein concentration of the supernatant by Pierce BCA protein assay. The minimum quantity for LC–MS/MS analysis is 20 μg of protein per sample. The extracts from each sample (50 μg protein) were mixed with acetone at a volume ratio of 1:4, then precipitated at − 20 °C for 2 h, and centrifuged at 20 000 g for 10 min. The supernatant was poured and the precipitant was washed twice more with acetone. Subsequently, each protein sample (20 μg protein) was reduced by DTT (60 min, 55 °C) and free cysteines alkylated with IAA ( 30 min, 25 °C in the dark). After these procedures, protein samples were loaded into 10 kDa ultrafiltration tubes, washed three times with 50 mM NH_4_HCO_3_. Samples were incubated with trypsin overnight at 37 °C. Digested peptides were transferred into a C18 peptide clean-up column, washed by Solvent A (0.1% FA in water) and eluted with elution buffer (60% ACN and 40% FA in water). Clean peptides were finally concentrated and dried in a SpeedVac (Eppendorf). All the blood samples were approved by the Human Ethics Review Committee of Science and Technology, Shanghai Jiao Tong University according to the Chinese regulation.

### LC–MS/MS analysis

Samples were analyzed on Q Exactive HF-X mass spectrometer (Thermo Fisher Scientific) with nano spray flex ion source and Thermo Scientific™ EASYnLCTM 1200 integrated ultra high-pressure nano HPLC system. The purified and dried peptides (500 ng) were re-dissolved in Solvent A and then automatically injected and loaded onto the trap column (75 μm × 2 cm; particle size, 3 μm; pore size, 100 Å; Thermo Fisher Scientific) at a flow rate of 2 μL min^−1^ (max pressure 500 bar). After 5 min, the peptides were eluted from the trap column and separated on the analytical column (75 μm × 25 cm; particle size, 2 μm; pore size, 100 Å; Thermo Fisher Scientific) by a gradient ranging from 8 to 100% of ACN mobile phase at 300 nL min^−1^ flow rate for 120 min.

### Label-free based protein identification and quantification

The acquired MS raw data were loaded to Proteome Discoverer^©^ (version 2.4, Thermo Scientific) software for label-free quantification. The database search was specified by trypsin as enzyme for digestion and peptides with up to two missed cleavages were included. The data exported from Proteome Discoverer was analyzed using Excel^©^ software. The normalized abundance for proteins and peptides were used for subsequent statistical analysis. Missing value and coefficient of variation (CV) value were mainly used for MS performance quality control. *k*-nearest neighbors algorithm was used for the missing value imputation method. The CV value was used to evaluate the dispersion of the replicates within one group, and proteins with CV ≤ 0.3 were considered reliable here. In addition, *P*-value was adjusted by Benjamin and Hochberg (BH, 1995) method. The normalized abundance ratio of CDS-PMo_12_@PVP_1_/CDS-PMo_12_@PVP_0_ was used for the calculation the FC. Differentially adsorption proteins were identified according to the following two criterions: *P*-value < 0.01 and FC > 2 and FC < 0.5.

## Supplementary Information

**Additional** **file** **1**: **Figure** **S1** TEM images of the CDS-PMo_12_@PVP_0_ NPs. **Figure** **S2** Size distributions of CDS-PMo_12_@PVP_x_ (x=0~1) NPs. (a) CDS-PMo_12_@PVP_0_, (b) CDS-PMo_12_@PVP_0.05_, (c) CDS-PMo_12_@PVP_0.1_, (d) CDS-PMo_12_@PVP_0.25_, (e) CDS-PMo_12_@PVP_0.5_ and (f) CDS-PMo_12_@PVP_1_. **Figure** **S3** FTIR spectra of the PVP, H_3_PMo_12_O_30_, CDS-PMo_12_@PVP_0,_ CDS-PMo_12_@PVP_0.5,_ and CDS-PMo_12_@PVP_1_ NPs. **Figure** **S4** Optimized preparation of absorbent. (a) Adsorption time, (b) pH, (c) temperature and (d) ionic strength of B-R buffer on the adsorption efficiency of BSA, Hb, and Cyt-C. Protein solution: 100 μg/mL, 1.0 mL; CDS-PMo_12_@PVP_0_: 5.0 mg. **Figure** **S5** Fluorescence emission spectra of (a-b) Cyt-C (d-e) Hb and (g-h) BSA decrease with the increasing amount of CDS-PMo_12_@PVP_0_ NPs at 298 K and 310 K. the maximum fluorescence intensity of (c) Cyt-C, (f) Hb and (i) BSA decrease in the presence of CDS-PMo_12_@PVP_0_ NPs (0-15 µM) at 298 K and 310 K, respectively. **Figure** **S6** Fluorescence emission spectra of (a-b) Cyt-C (d-e) Hb and (g-h) BSA decrease with the increasing amount of CDS-PMo_12_@PVP_1_ NPs at 298 K and 310 K. the maximum fluorescence intensity of (c) Cyt-C, (f) Hb and (i) BSA decrease in the presence of CDS-PMo_12_@PVP_1_ NPs (0-15 µM) at 298 K and 310 K, respectively.** Figure S7** Stern-Volmer plot derived from the fluorescence emission spectrum of the (a) Cyt-C, (b) Hb, and (c) BSA interaction with PMo_12_ NPs. The Modified Stern-Volmer plot of (d) Cyt-C, (e) Hb, and (f) BSA interaction with PMo_12_ NPs. The binding logarithmic graph of (g) Cyt-C, (h) Hb, and (i) BSA interaction with PMo_12_ NPs at 298 K and 310 K, respectively. **Figure** **S8** The heatmap of 76 differential proteins of CDS-PMo_12_@PVP_1_ comparing to CDS-PMo_12_@PVP_0_, as identified by LC-MS/MS. **Table** **S1** The top ten up-regulated and down-regulated proteins of 76 differential proteins.
