# Supplementary material for: Poly (N-vinylpyrrolidone) modification mitigates plasma protein corona formation on phosphomolybdate-based nanoparticles
Source: J Nanobiotechnology. 2021 Dec 23;19:445. doi: 10.1186/s12951-021-01140-8 (PMC8697440; doi:10.1186/s12951-021-01140-8)

Use the "Insert Citation" button to add citations to this document.

**Supporting Information**

**Poly (N-vinylpyrrolidone) Modified Phosphomolybdate-based Nanoparticles Mitigates Plasma Protein Corona Formation for Nanodrug Design**

*Youyi Yu, Behafarid Ghalandari, Guangxia Shen, Liping Wang, Xiao Liu,Aiting Wang, Sijie Li, Haiyang Xie* and Xianting Ding**

State Key Laboratory of Oncogenes and Related Genes, Institute for Personalized Medicine, School of Biomedical Engineering, Shanghai Jiao Tong University, Shanghai, 200030, China.

**Table of Contents**

1. **Experimental details**
2. **Electron microscopy analysis**
3. **Size statistics analysis**
4. **FTIR spectra**
5. **The model proteins adsorption conditions**
6. **Fluorescence emission spectra of proteins with CDS-PMo_12_@PVP_0_ NPs**
7. **Fluorescence emission spectra of proteins with CDS-PMo_12_@PVP_1_ NPs**
8. **Stern-Volmer plot, Modified Stern-Volmer plot and the binding logarithmic graph**
9. **The heatmap of differential proteins**
10. **The top ten up-regulated and down-regulated proteins.**

**1. Experimental details**

**Synthesis of cesium dodecylsulfate (CDS).**

A saturated water solution of sodium dodecylsulfate (1 g, 3.5 mmole) was treated with 5% HCl (a few drops) to get pH ~ 0. This mixture treated with a saturated water solution of cesium carbonate (0.75 g, 5 mmol) and white waxy solid CDS precipitated upon stirring. The product collected by vacuum filtration and dried at temperature (0.96 g, 2.5 mmol, 72% yield).

**Synthesis of CDS-PMo_12_@PVP_0_ NPs**

An aqueous solution of H_3_PMo_12_O_40_^.^3H_2_O (0.01 M, 200 μL) was added dropwise to an aqueous solution of CDS (0.01M, 500 μL) at 25 °C under vigorous magnetic stirring, produced a pale-yellow precipitate CDS-PMo_12_@PVP_0_ NPs immediately_._

**Synthesis of CDS-PMo_12_@PVP_x_(x=0.05~1) NPs**

A mixture of 200 µL of a 0.01 M H_3_PMo_12_O_40_ water solution and 800 µL of different concentration (0.05 g/L, 0.1 g/L, 0.25 g/L, 0.5 g/L, and 1 g/L) of a poly (N-vinylpyrrolidone) (PVP) aqueous, then, the mixture was added to 500 µL 0.01 M water solution of CDS, under vigorous magnetic stirring. The formation process of pale-yellow precipitate slowed down (0.5-24 h). The synthesized nanoparticles named as CDS-PMo_12_@PVP_0.05,_ CDS-PMo_12_@PVP_0.1_, CDS-PMo_12_@PVP_0.25_, CDS-PMo_12_@PVP_0.5_ and CDS-PMo_12_@PVP_1_, respectively.

**2. Electron microscopy analysis**

**Sample preparation for Transmittance electron microscopy analysis**

Nanoparticles deposition for TEM analysis. Freshly synthesized nanoparticles suspended in ddH_2_O and a 3 µL aliquot was deposited on a thin carbon film and dried at room temperature.


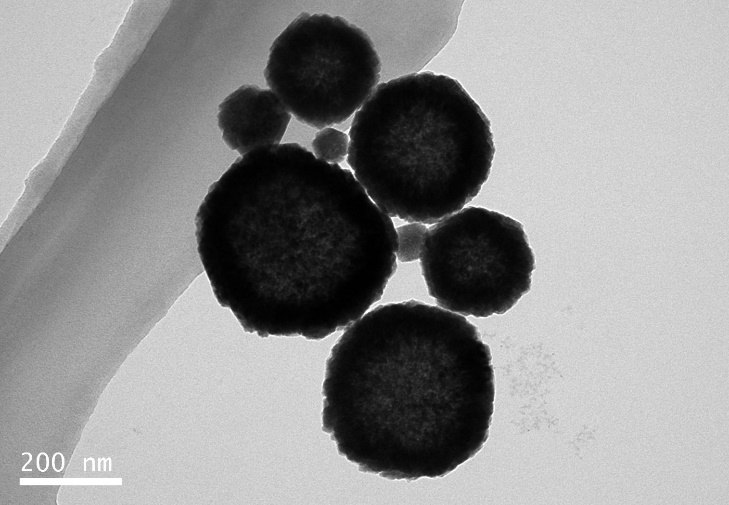


**Figure S1** TEM images of the CDS-PMo_12_@PVP_0_ NPs.

1. **Size statistics analysis**


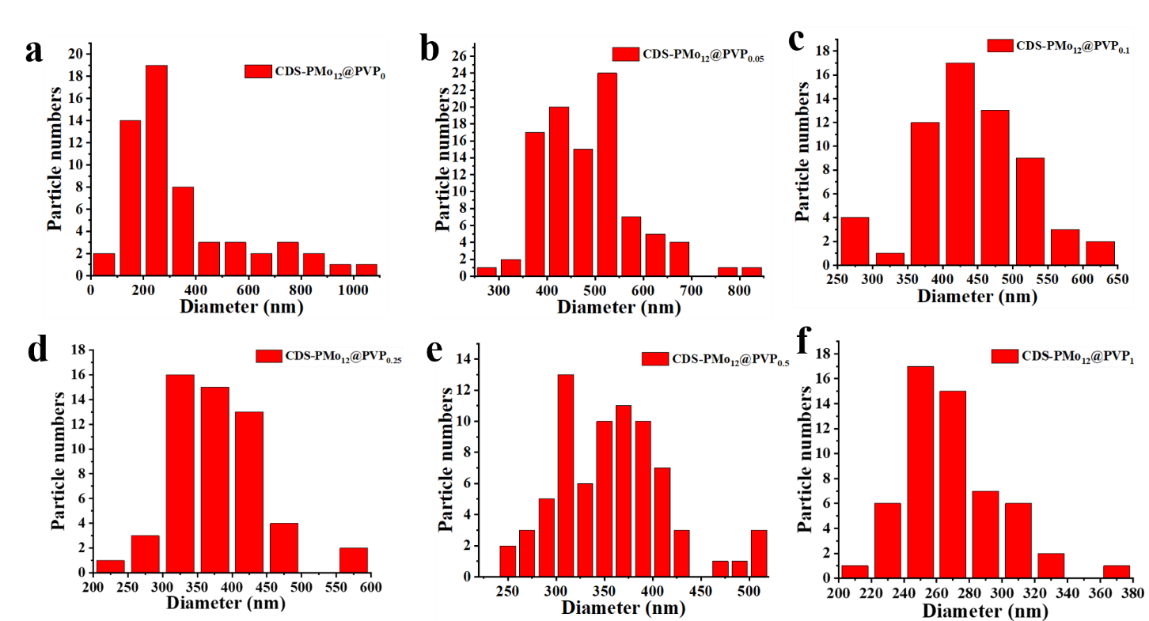


**Figure S2** Size distributions of CDS-PMo_12_@PVP_x_ (x=0~1) NPs. (a) CDS-PMo_12_@PVP_0_, (b) CDS-PMo_12_@PVP_0.05_, (c) CDS-PMo_12_@PVP_0.1_, (d) CDS-PMo_12_@PVP_0.25_, (e) CDS-PMo_12_@PVP_0.5_ and (f) CDS-PMo_12_@PVP_1_.

1. **FTIR spectra**


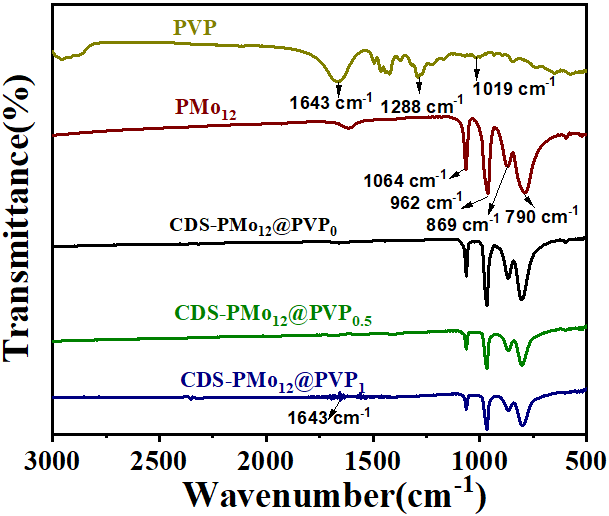


**Figure S3** FTIR spectra of the PVP, H_3_PMo_12_O_30_, CDS-PMo_12_@PVP_0,_ CDS-PMo_12_@PVP_0.5,_ and CDS-PMo_12_@PVP_1_ NPs.

1. **The model proteins adsorption conditions**


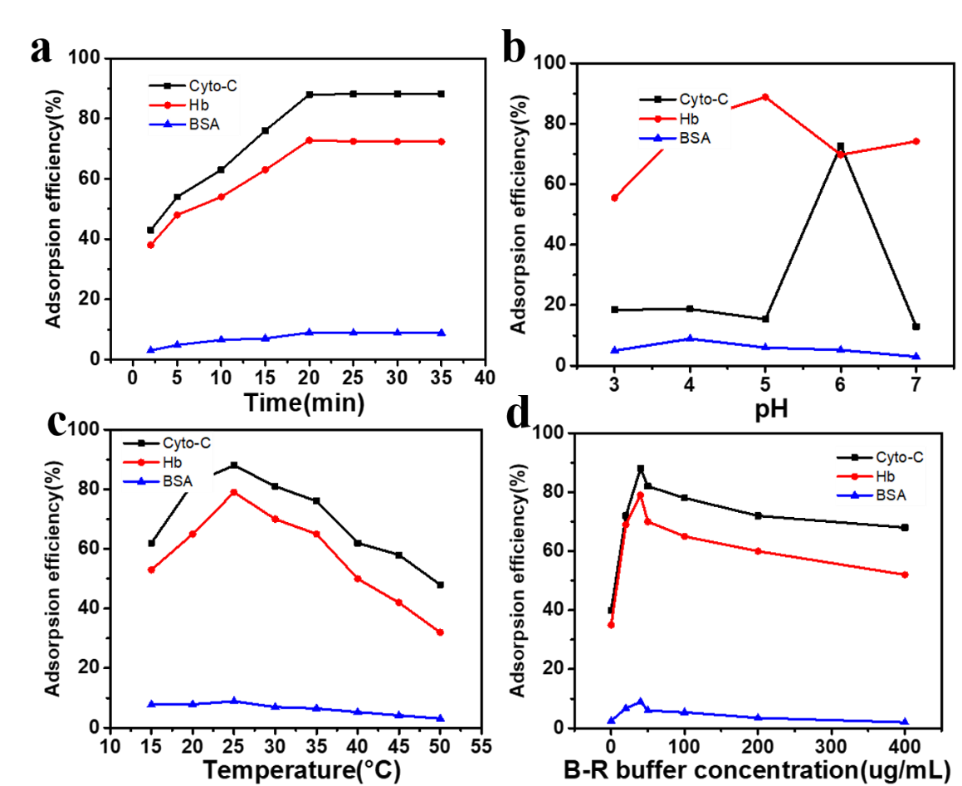


**Figure S4** Optimized preparation of absorbent. (a) Adsorption time, (b) pH, (c) temperature and (d) ionic strength of B-R buffer on the adsorption efficiency of BSA, Hb, and Cyt-C. Protein solution: 100 μg/mL, 1.0 mL; CDS-PMo_12_@PVP_0_: 5.0 mg.

1. **Fluorescence emission spectra of proteins with CDS-PMo_12_@PVP_0_ NPs**


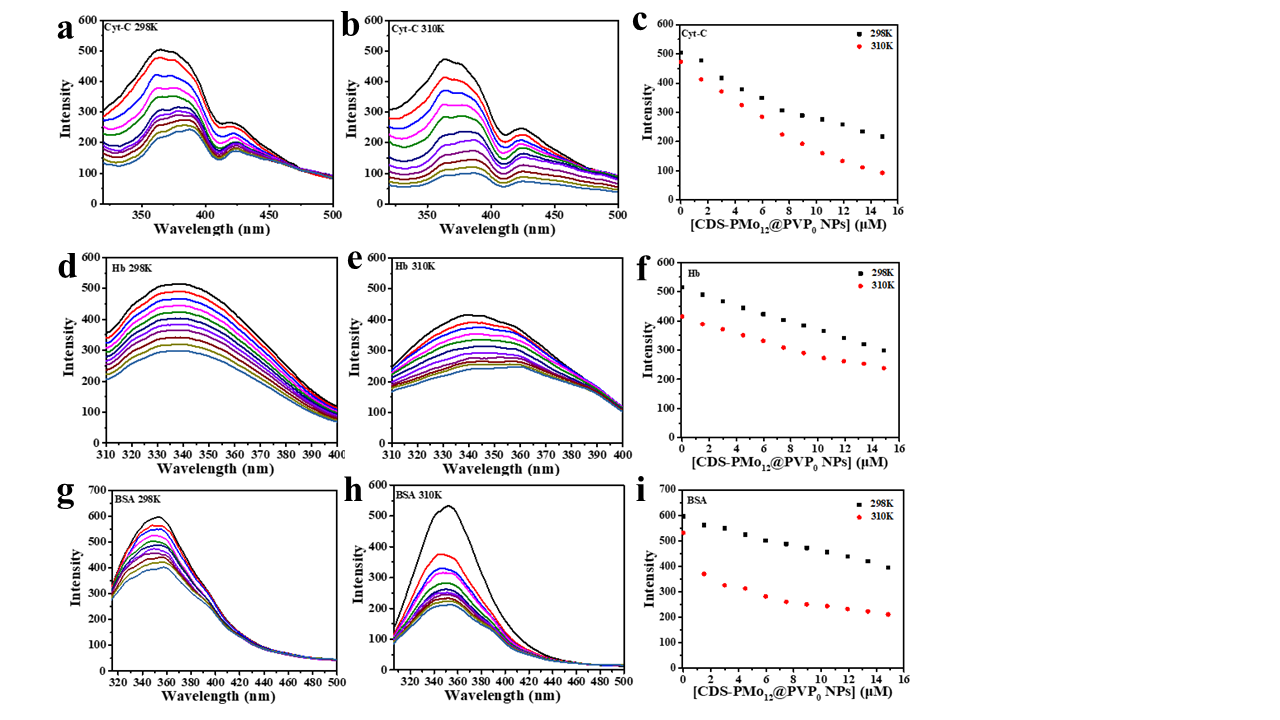


**Figure S5** Fluorescence emission spectra of (a-b) Cyt-C (d-e) Hb and (g-h) BSA decrease with the increasing amount of CDS-PMo_12_@PVP_0_ NPs at 298 K and 310 K. the maximum fluorescence intensity of (c) Cyt-C, (f) Hb and (i) BSA decrease in the presence of CDS-PMo_12_@PVP_0_ NPs (0-15 µM) at 298 K and 310 K, respectively.

1. **Fluorescence emission spectra of proteins with CDS-PMo_12_@PVP_1_ NPs**

**
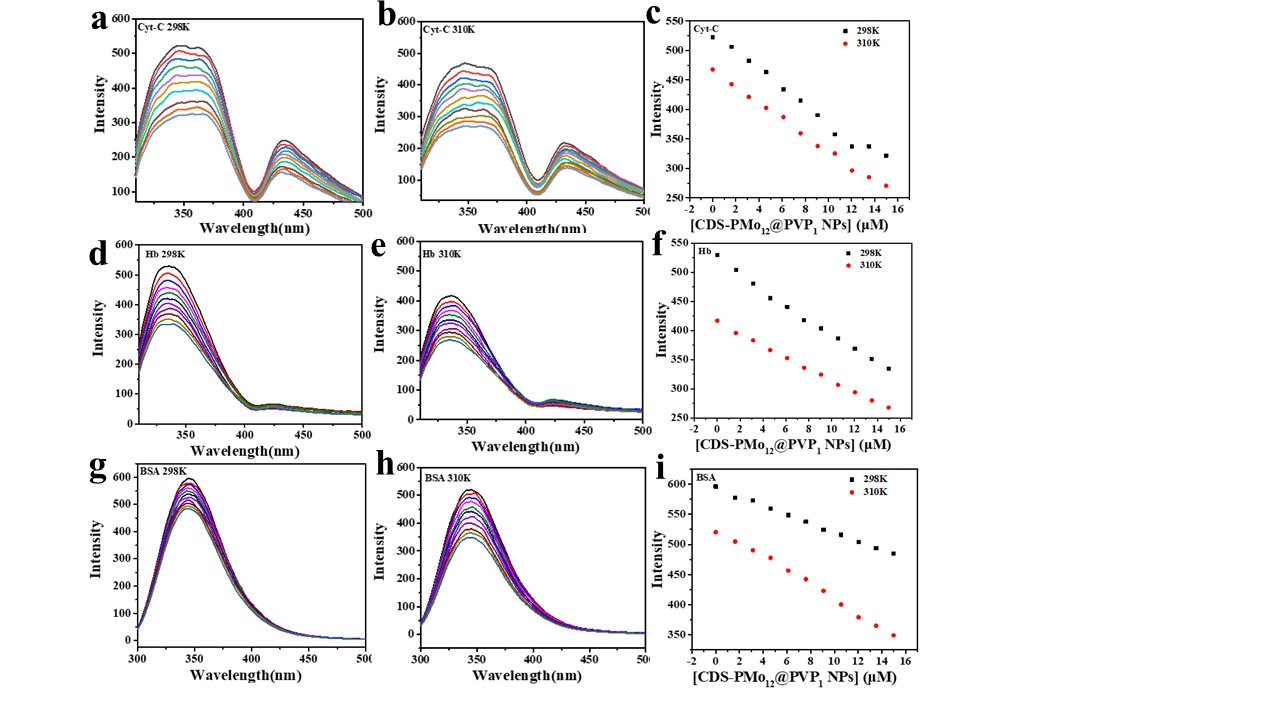
**

**Figure S6** Fluorescence emission spectra of (a-b) Cyt-C (d-e) Hb and (g-h) BSA decrease with the increasing amount of CDS-PMo_12_@PVP_1_ NPs at 298 K and 310 K. the maximum fluorescence intensity of (c) Cyt-C, (f) Hb and (i) BSA decrease in the presence of CDS-PMo_12_@PVP_1_ NPs (0-15 µM) at 298 K and 310 K, respectively.

1. **Stern-Volmer plot, Modified Stern-Volmer plot and the binding logarithmic graph**


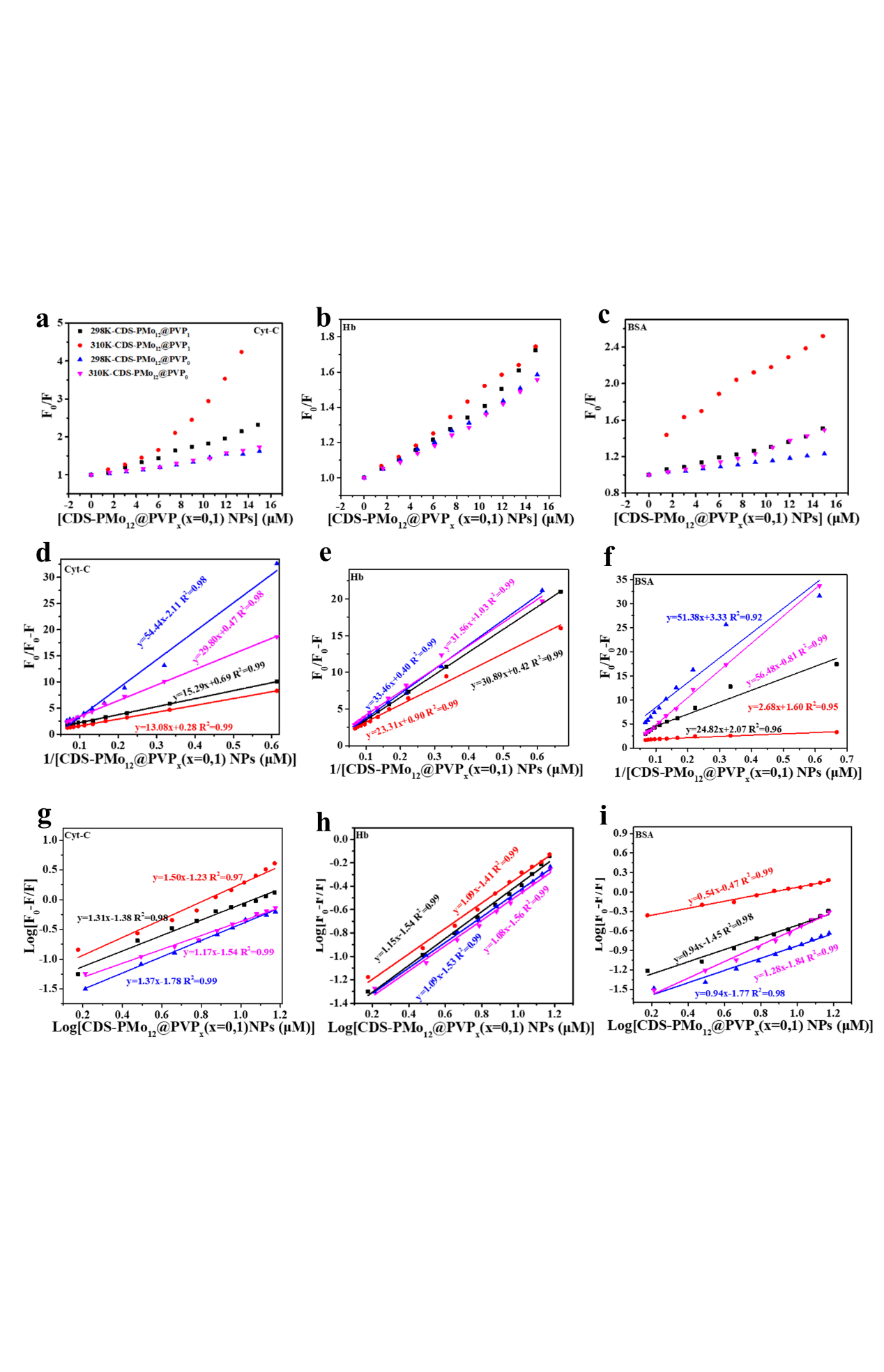


Figure S7 Stern-Volmer plot derived from the fluorescence emission spectrum of the (a) Cyt-C, (b) Hb, and (c) BSA interaction with PMo_12_ NPs. The Modified Stern-Volmer plot of (d) Cyt-C, (e) Hb, and (f) BSA interaction with PMo_12_ NPs. The binding logarithmic graph of (g) Cyt-C, (h) Hb, and (i) BSA interaction with PMo_12_ NPs at 298 K and 310 K, respectively.

1. **The heatmap of differential proteins**


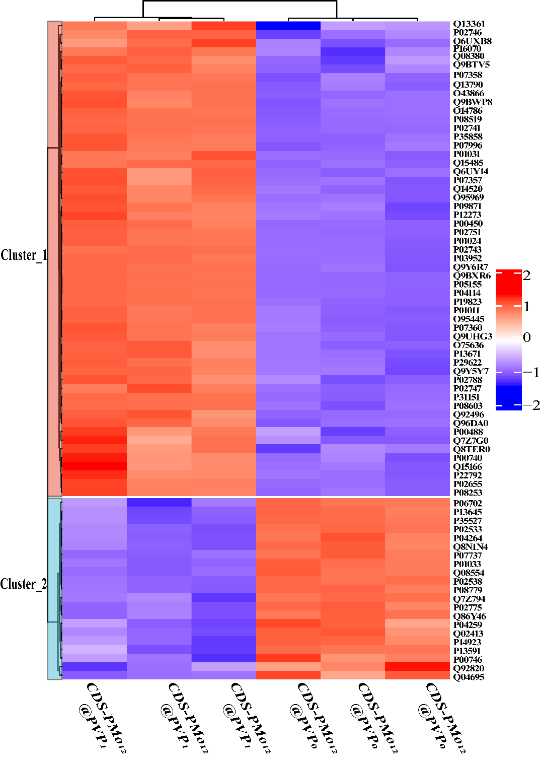


**Figure S8** The heatmap of 76 differential proteins of CDS-PMo_12_@PVP_1_ comparing to CDS-PMo_12_@PVP_0_, as identified by LC-MS/MS.

1. **The top ten up-regulated and down-regulated proteins.**

**Table S1** The top ten up-regulated and down-regulated proteins of 76 differential proteins.


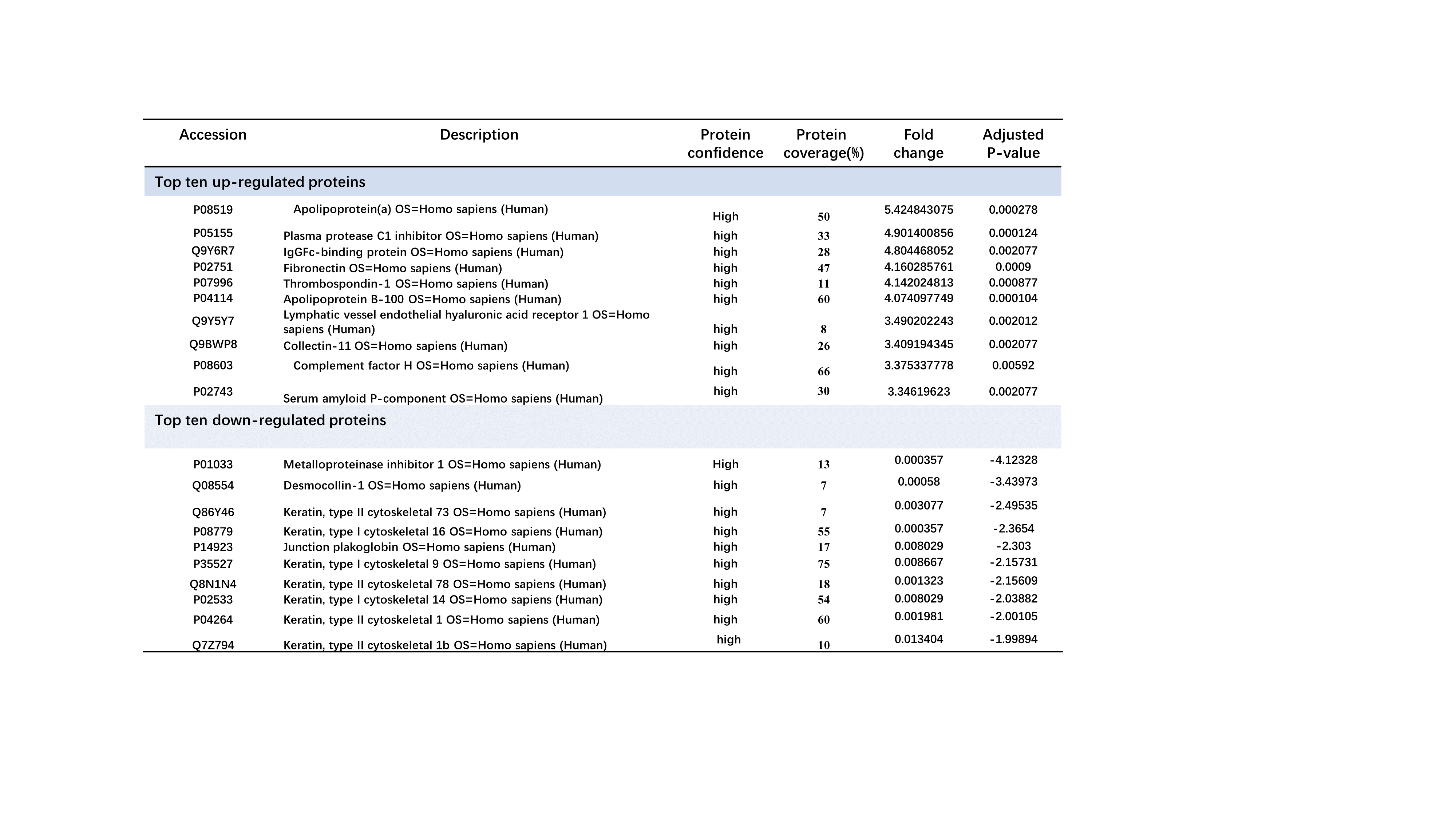

Supplement: Supplementary file 1 — Additional file 1: Figure S1 TEM images of the CDS-PMo12@PVP0 NPs. Figure S2 Size distributions of CDS-PMo12@PVPx (x=0~1) NPs. (a) CDS-PMo12@PVP0, (b) CDS-PMo12@PVP0.05, (c) CDS-PMo12@PVP0.1, (d) CDS-PMo12@PVP0.25, (e) CDS-PMo12@PVP0.5 and (f) CDS-PMo12@PVP1. Figure S3 FTIR spectra of the PVP, H3PMo12O30, CDS-PMo12@PVP0, CDS-PMo12@PVP0.5, and CDS-PMo12@PVP1 NPs. Figure S4 Optimized preparation of absorbent. (a) Adsorption time, (b) pH, (c) temperature and (d) ionic strength of B-R buffer on the adsorption efficiency of BSA, Hb, and Cyt-C. Protein solution: 100 μg/mL, 1.0 mL; CDS-PMo12@PVP0: 5.0 mg. Figure S5 Fluorescence emission spectra of (a-b) Cyt-C (d-e) Hb and (g-h) BSA decrease with the increasing amount of CDS-PMo12@PVP0 NPs at 298 K and 310 K. the maximum fluorescence intensity of (c) Cyt-C, (f) Hb and (i) BSA decrease in the presence of CDS-PMo12@PVP0 NPs (0-15 µM) at 298 K and 310 K, respectively. Figure S6 Fluorescence emission spectra of (a-b) Cyt-C (d-e) Hb and (g-h) BSA decrease with the increasing amount of CDS-PMo12@PVP1 NPs at 298 K and 310 K. the maximum fluorescence intensity of (c) Cyt-C, (f) Hb and (i) BSA decrease in the presence of CDS-PMo12@PVP1 NPs (0-15 µM) at 298 K and 310 K, respectively. Figure S7 Stern-Volmer plot derived from the fluorescence emission spectrum of the (a) Cyt-C, (b) Hb, and (c) BSA interaction with PMo12 NPs. The Modified Stern-Volmer plot of (d) Cyt-C, (e) Hb, and (f) BSA interaction with PMo12 NPs. The binding logarithmic graph of (g) Cyt-C, (h) Hb, and (i) BSA interaction with PMo12 NPs at 298 K and 310 K, respectively. Figure S8 The heatmap of 76 differential proteins of CDS-PMo12@PVP1 comparing to CDS-PMo12@PVP0, as identified by LC-MS/MS. Table S1 The top ten up-regulated and down-regulated proteins of 76 differential proteins. [file 12951_2021_1140_MOESM1_ESM.docx]
